# Finding the Right Plugin: Mosquitoes Have the Answer

**DOI:** 10.1371/journal.pbio.1000273

**Published:** 2009-12-22

**Authors:** Tracey Chapman

**Affiliations:** School of Biological Sciences, University of East Anglia, Norwich, Norfolk, United Kingdom; United States of America

## Abstract

The intriguing composition and function of mating plugs formed when mosquites mate provides a new understanding of the reproductive biology of this important pest and a window through which to view evolution in action.

The chemical complexity of the male ejaculate is truly extraordinary and every bit as remarkable as the most extravagant male plumage and courtship displays. This protein-rich seminal fluid delivers chemical messages that can enter the female brain and modify behaviour, stimulate muscle contractions in, and change the appearance of the female reproductive tract, and cause females to release reproductive hormones. In fact, these chemicals can collectively modify almost all aspects of female reproductive behaviour and physiology (e.g., [1]–[3]).

Our understanding of the role of the non-sperm constituents of insect ejaculates has been hugely enriched by classic work in *Drosophila melanogaster*. In a series of papers dating from the 1980s, the Wolfner and Kubli research groups, and more recently the Swanson laboratory, have identified over 100 proteins and peptides synthesised in the male reproductive system and transferred to females during mating (e.g., [4]–[9]). Increasingly the functional significance of these substances is being shown, with many having significant effects on fitness [10]–[12]. A more recent focus has been on identifying seminal fluid proteins in species of agricultural and medical importance [13],[14], and excellent papers have recently been published on the identification of seminal fluid proteins in the mosquito vectors of dengue/yellow fever and of malaria [15],[16].

## Isolation of Seminal Fluid Proteins in Mosquitoes

The history of the study of the seminal fluid proteins in mosquitoes goes back at least 40 years. Following the great tradition for physiological experiments in the 1960s and 70s, accessory glands were implanted or their extracts injected into females, and the effects on female receptivity or egg laying examined [17]–[22]. In addition, mating or accessory gland extract injection in the yellow fever vector *Aedes aegypti* is also reported to affect flight (e.g., [23]), responses to host cues (e.g., [20]), and digestion of blood meals (e.g., [24]). Following on from this—dare I say it—seminal work, accessory gland substances in *Ae. aegypti* [19],[22] and in *Culex tarsalis* were described [25],[26], but never fully characterised. However, following this heyday of careful physiological experiments, large-scale characterisations of mosquito seminal fluid proteins were lacking until the application over the last few years of genomic and proteomic techniques, such as mass spectrometry (MS). However, for the potential of techniques like MS to be realised comprehensively, it is necessary to have a sequenced genome together with accurate protein predictions and annotations, or at least a thoroughly sequenced library of expressed sequences from the tissues of interest. Hence, it was the sequencing of mosquito genomes that has led to the recent increase in large-scale approaches to studying mosquito reproduction. These new methods have allowed the simultaneous identification of tens of seminal fluid proteins and have revolutionised this exciting and now rapidly advancing field. The advantages are easy to see, set against the laborious and hugely time-consuming procedures of identifying individual seminal fluid protein substances by purification of extracts and separation by high-performance liquid chromatography.

It is true, however, that it is often easier to identify seminal fluid proteins than to find out what they do. Although it is possible to gain significant insight through comparisons of sequences and the discovery of functional domains, conclusive proof of function usually depends upon the ability to manipulate the production or expression of the protein or peptide involved. This can mean creating and using loss-of-function mutants, post-transcriptional silencing, or over-expression. There may often be limited opportunities to apply these techniques effectively in non-model systems. Functional tests have revealed the truly remarkable diversity of phenotypes that are influenced by seminal fluid proteins. We know from work in *Drosophila*, for example, that seminal fluid proteins cause females to lay more eggs [4], to ovulate at a higher rate [27], to eat more [28] (which may cause a reduction in “sleep” like inactivity [29]), to synthesise Juvenile Hormone [30], and to produce more antimicrobial peptides [31]. Seminal fluid proteins also play an essential role in ensuring sperm are stored and retained [32]–[34] and are associated with a male's success in sperm competition (e.g., [35]–[37]).

## Functions of Seminal Fluid Proteins: Formation of the Mating Plug

One important function of seminal fluid is to form “mating plugs” [38], the very variable structures that are often made within the female reproductive tract during or shortly after mating (Box 1). Mating plugs are reported widely from insects to mammals, come in many shapes and sizes, and have three suggested functions: (i) to prevent remating, either by physically blocking subsequent mating attempts or releasing chemical cues that prevent females from remating; (ii) to help sperm storage or to prevent sperm loss from the female reproductive tract; and (iii) to act as a visible signal of female mating status [38]–[41]. Mating plugs can be large and elaborate, ephemeral and simple, and their constituents vary enormously—from fats to the male genitalia themselves [39],[42].

**Box 1. Evolution and Function of Mating Plugs across Invertebrate and Vertebrate Taxa**

The structure of mating plugs across vertebrate and invertebrate taxa is fascinatingly variable, and structural components of plugs have been identified across vertebrate and invertebrate taxa (e.g. in primates including humans, rodents, *Caenorhabditis elegans*, Diptera, Lepidoptera, and Hymenoptera). Mating plugs often comprise proteins, lipids, or mucins, but in invertebrates they can even be formed from part of the male's genitalia, which may result in permanent damage to the male or even death (summarised in [43]). In functional terms, some broad similarities are apparent. First, in plugs formed from the aggregation of chemical constituents, rather than actual body parts, there is usually cross-linkage of mating plug components in order to achieve solidification or coagulation, followed by a process of dissolution. Second, where studied, some of the genes encoding mating plug constituents show elevated rates of evolutionary change in species where sperm competition is strong, supporting the notion that plugs in some species play a role in male–male (sperm) competition.

The mating plugs of primates and rodents contain products from a family of rapidly evolving seminal vesicle-transcribed (REST) genes [56]. These include the semenogenins of primates and the seminal vesicle (Svs) genes of rodents. The nature of the cross-linking that provides coagulation of the plugs is in both cases now known. These genes have been subject to evolutionary analysis [57]–[59]. In addition, the evolution of the polymorphism that in *C. elegans* controls whether males do or do not transfer mating plugs has also been revealed [60]. Semenogenin 2 (SEMG2) is a structural component of primate mating plugs that becomes cross-linked during coagulation and cleaved during plug dissolution by a component of the male prostate, kallikrein 3 (or prostate-specific antigen). The evolution of *SEMG2* appears elevated among promiscuous primate species, supporting a possible role in sperm competition [57]–[59]. Consistent with this finding, in rodents, the evolution of *seminal vesicle 2* (*Svs2*), which encodes a major component of the mating plug, also appears to show elevated positive selection in species experiencing strong sperm competition [58]. Another interesting approach that supports the role of mating plugs in sperm competition comes from *C. elegans*, in which the genetic basis of the polymorphism for mating plug formation has been identified [60]. The lack of the ability to form a mating plug is essentially a loss-of-function mutation that appears to have spread because of the concomitant origin of hermaphroditism within this group. In sum, is clear that there is fundamental evolutionary insight to be revealed by the powerful approach of examining the function and evolution of genes encoding mating plug components.

There is good evidence that plugs sometimes function in reducing female remating, and this can be a temporary effect as in *Drosophila* (e.g., [43]) or a permanent switch as the bumblebee *Bombus terrestris* [44]. Hence, the plug can potentially be a physical chastity belt in the sense of the first known historical description, “hard iron breeches …. closed at the front” [45], but can also serve its purpose through effects on female behaviour alone. The potential for plugs to be used as indicators of recent mating is presumably selected in the context of male–male competition to reduce the likelihood of female remating. Such signals are sometimes very obvious, for example, keepers of mice will know the utility of “plugging” as a reliable indicator of pregnancy. There is also evidence from flies that at least one seminal protein component of the mating plug can help sperm to be retained in storage [32],[38].

A new study by Rogers et al. published in this issue of *PLoS Biology* [46], capitalises on new genomic and proteomic techniques, sequence comparisons, and bioimaging techniques, and provides an excellent example of both the identification of mating plug components and of their functional significance in the malaria vector *Anopheles gambiae*. In recent work, this group reported the characterisation of seminal fluid proteins in this species [16]. However, until now it was not clear whether any of these proteins were actually transferred into females. This study makes several significant advances: it characterises the mating plug proteins in *An. gambiae* and identifies which of the components of these comes from males and which from females. Intriguingly, some female proteins become associated with the plug after its transfer from the male reproductive tract into the female atrium. The study is a real advance because, in addition to the proteomic identification and analysis of transferred proteins, the authors have also done a series of careful genetic and imaging experiments to understand the specific functions of two plug proteins. The team showed that coagulation of the plug is caused by cross-linking of a major component, suitably named “plugin”, by a specific transglutaminase, also expressed in the male reproductive tract accessory glands. This process turned out to be remarkably similar to the coagulation reported in mammalian ejaculates, and was tellingly absent, as predicted, in mosquito species that lack mating plugs. By injecting double-stranded RNA into adult males to target the transglutaminase, a significant level of knockdown was achieved. Importantly, males that produced reduced amounts of transglutaminase failed to form and transfer a plug to females. The vast majority of females mated to these males did not receive a plug and were not inseminated. Alongside this, it was also shown that the physical presence of the plug was not sufficient to prevent female remating. Hence, the evidence suggests that the primary function of the plug is to ensure that sperm are stored.

## Future Prospects

The new Rogers et al. study [46] used a powerful array of different approaches to analyse the function of mating plug proteins in *An. gambiae*. It provides important data for understanding the reproductive biology of this important pest and suggests potential new avenues for manipulating its reproductive biology. However, as with any good study, it also opens up many more questions than it answers. For example, it contributes to the general observation that both male and female factors are often required for the correct processing of ejaculates [47]. This explains why injection of male accessory gland extracts may sometimes fail to find biological effects even if they exist [48],[49], because they bypass this essential processing. The female proteins in the mating plug included proteases, and some are expressed at high levels in the atrium of virgin females and are permanently switched off by 24 h post mating [50]. This is consistent with the idea that female mosquitoes express a subset of atrial proteins for processing the mating plug within the first 24 h. The key question is, however, what are the female components of this there for—to correctly and efficiently process the plug, or to try to break it down? The ultimate reasons will be illuminating to discover. Together the findings suggest, as noted in *Drosophila*, that intimate and complex chemical interplay exists between males and females at mating [51].

*An. gambiae* males, as well as transferring the proteins described in the new study [46], also transfer the steroid hormone ecdysone [52]. Some species of Lepidoptera are reported to transfer the other major reproductive hormone, Juvenile Hormone [53]. The puzzle is why males of some species transfer reproductive hormones themselves, whereas others transfer the initiating signals that cause females to make them. There are physiologically significant questions regarding the compartmentalisation of seminal fluid protein synthesis within the male reproductive tract and of the separation in males and females of molecules required for a common purpose (e.g., the formation or processing of the mating plug). Similar patterns are reported in seminal fluid processing in *Drosophila* and mice and this physical separation of proteins/molecules within reproductive tracts might be a common strategy to efficiently coordinate seminal fluid functions. A general feature of reproductive biology that the Rogers et al. [46] study also supports is the finding of functional conservation across taxa, with identical classes of reproductive molecules being observed (proteases, protease inhibitors, lipases, cysteine-rich secretory proteins), despite different underlying mechanisms, i.e., these molecules being encoded by non-homologous genes [54],[55]. In addition, a particularly intriguing question is why some mosquitoes that mate only once form mating plugs while others do not.

Collectively, the study of insect reproductive systems is highlighting unexpected findings, such as almost unprecedented evolutionary lability in the genes encoding reproductive proteins [6],[9]. As such, these models offer a superb window through which to view evolution in action, to elucidate fundamental principles of evolutionary and reproductive biology, and to suggest how basic research can also provide essential knowledge in the development of new routes for insect control.
